# Elevated levels of enteric IgA in an unimmunised mouse model of Hyper IgM syndrome derived from gut-associated secondary lymph organs even in the absence of germinal centres

**DOI:** 10.3389/fcimb.2023.1172021

**Published:** 2023-06-29

**Authors:** Felipe Hernandez-Cazares, Raul Antonio Maqueda-Alfaro, Catalina Lopez-Saucedo, Jesus Martinez-Barnetche, Juan Carlos Yam-Puc, Sergio Estrada-Parra, Leopoldo Flores-Romo, Teresa Estrada-Garcia

**Affiliations:** ^1^ Cell Biology Department, CINVESTAV-IPN, Ciudad de México, Mexico; ^2^ Department of Molecular Biomedicine, CINVESTAV-IPN, Ciudad de México, Mexico; ^3^ Centro de Investigación Sobre Enfermedades Infecciosas, Instituto Nacional de Salud Pública, Cuernavaca, Morelos, Mexico; ^4^ MRC Toxicology Unit, University of Cambridge, Cambridge, United Kingdom; ^5^ Departamento de Inmunología, Escuela Nacional de Ciencias Biológicas, Instituto Politécnico Nacional, Ciudad de México, Mexico

**Keywords:** C57-CD40L-deficient mice, gut-associated secondary-lymphoid-organs, B cell populations, IgA-production, unimmunised

## Abstract

**Introduction:**

Patients with Human Hyper IgM syndromes (HIGM) developed pulmonary and gastrointestinal infections since infancy and most patients have mutations in the CD40 ligand (CD40L) gene. Most HIGM patients compared to healthy subjects have higher/similar IgM and lower IgG, and IgA serum concentrations but gut antibody concentrations are unknown. CD40L on activated T-cells interacts with CD40 on B-cells, essential for the formation of germinal centres (GCs) inside secondary lymphoid organs (SLOs), where high-affinity antibodies, long-lived antibody-secreting plasma cells, and memory B-cells, are produced. C57BL6-CD40 ligand deficient mice (C57BL6-*cd40l* ^−/−^), are a model of HIGM, because serum immunoglobulin concentrations parallel levels observed in HIGM patients and have higher faecal IgA concentrations. In mice, TGFβ and other cytokines induce IgA production.

**Aims:**

To compare and evaluate B-cell populations and IgA-producing plasma cells in peritoneal lavage, non-gut-associated SLOs, spleen/inguinal lymph nodes (ILN), and gut-associated SLOs, mesenteric lymph nodes (MLN)/Peyer´s patches (PP) of unimmunised C57BL6-*cd40l* ^−/−^ and C57BL6-wild-type (WT) mice.

**Material and methods:**

Peritoneal lavages, spleens, ILN, MLN, and PP from 8-10 weeks old C57BL6-*cd40l* ^−/−^ and WT mice, were obtained. Organ cryosections were analysed by immunofluorescence and B-cell populations and IgA-positive plasma cell suspensions by flow cytometry.

**Results:**

In unimmunised WT mice, GCs were only observed in the gut-associated SLOs, but GCs were absent in all C57BL6-*cd40l* ^−/−^ SLOs. PP and MLN of C57BL6-*cd40l* ^−/−^ mice exhibited a significantly higher number of IgA-producing cells than WT mice. In the spleen and ILN of C57BL6-*cd40l^−^* ^/−^ mice IgA-producing cells significantly decreased, while IgM-positive plasma cells increased. C57BL6-*cd40l* ^−/−^ B-1 cells were more abundant in all analysed SLOs, whereas in WT mice most B-1 cells were contained within the peritoneal cavity. C57BL6-*cd40l* ^−/−^ B-cells in MLN expressed a higher TGFβ receptor-1 than WT mice. Mouse strains small intestine microvilli (MV), have a similar frequency of IgA-positive cells.

**Discussion:**

Together our results confirm the role of PP and MLN as gut inductive sites, whose characteristic features are to initiate an IgA preferential immune response production in these anatomical sites even in the absence of GCs. IgA antibodies play a pivotal role in neutralising, eliminating, and regulating potential pathogens and microorganisms in the gut.

## Introduction

Patients with Human Hyper IgM syndrome (HIGM), usually develop symptoms in infancy and are prone to pulmonary and gastrointestinal infections and complications (Moazzami et al., 2019). HIGM syndrome was first described back in 1961 and was first molecularly characterised as a mutation in the CD40 ligand (CD40L) gene in 1992 (Cabral-Marques et al., 2014). Patients are characterised by presenting with higher or similar concentrations of serum IgM but lower IgG, IgA, or absent concentrations of IgE compared to healthy subjects, accordingly, serum IgG immunoglobulin replacement is an effective host treatment for decreasing chronic infections among these patients (Davies and Thrasher, 2010; Yazdani et al., 2019). CD40L deficiency ranks as the 28th most frequent type of primary immunodeficiency disorder plus defects to the CD40L (48-70%) molecule are the most common cause of HIGM syndromes worldwide (Yazdani et al., 2019).

The CD40L also known as CD154, is a type II transmembrane protein expressed mainly by activated T cells and platelets and its expression is also induced under inflammatory conditions on basophils, monocytes, natural killers, and mast cells (Meng et al., 2018). In contrast, CD40 is a constitutively expressed receptor that initially was characterised on B-cells and also expressed on dendritic cells (DCs), monocytes, platelets, macrophages, and many non-hematopoietic cells (myofibroblasts, fibroblasts, epithelial, and endothelial cells) (Elgueta et al., 2009). The wide expression of this costimulatory pair of molecules indicates the pivotal roles they play in different cellular immune processes, e.g., CD40 signalling of B-cells by CD40L on activated T cells is essential for the formation of Germinal Centres (GCs) inside Secondary Lymphoid Organs (SLOs). GCs are highly specialized microstructures comprised of B-cells undergoing clonal expansion and affinity maturation (Klein and Dalla-Favera, 2008; Victora and Nussenzweig, 2022). Within GCs, B-cells undergo the Darwinian process of somatic diversification, and affinity-driven selection of immunoglobulins producing high-affinity (IgG, IgA, and IgE) antibodies, long-lived antibody-secreting plasma cells, and memory B-cells, that together are essential for effective humoral immunity, including the elimination of pathogens and protecting the host against reinfections (Klein and Dalla-Favera, 2008; Victora and Nussenzweig, 2022).

Focusing on intestinal antibody production during homeostasis, it is well known that it is dominated by IgA production, albeit intestinal B-cell IgG class switching also occurs although at a low level (Fleming et al., 2022). Furthermore, it has been demonstrated that within mice MLN and PP commensal-reactive IgG2b and IgG3 responses. These responses are largely generated independently of T cells (TI), requiring B-cell Toll-like receptor (TLR) signalling, therefore, gut microbiota-induced IgG antibodies predominantly IgG2b and IgG3, it is well known that TI IgG3 responses in mice predominantly target microorganisms’ carbohydrate antigens (Pone et al., 2010; Rawlings et al., 2012; Koch et al., 2016; Fleming et al., 2022).

Additionally, to the conventional B-2 cell population, there is another population of B-cells known as B-1 cells that produce antibodies that do not require the signalling between CD40 and CD40L on B-cells and on activated T cells, respectively. B-1 cells are more abundant in the pleural and peritoneal cavities, anatomical regions continuously exposed to microorganisms (Kroese et al., 1989; Gao et al., 2012). Therefore, B-1 cells migrate to both SLOs and intestinal lamina propria spontaneously secreting natural IgM, IgA, and IgG3 antibodies, in the absence of exogenous immunization, mainly against non-protein antigens including those present on pathogenic microbiota microorganisms (Meyer-Bahlburg, 2015; Prieto and Felippe, 2017; Castro-Dopico and Clatworthy, 2019). B-1 cells switch to IgA expression more readily than other B-cell populations (B-2) when exposed to certain soluble cytokines e.g., transforming growth factor beta (TGFβ), a proliferation-inducing ligand (APRIL), B-cell activating factor (BAFF) or bacterial molecules like lipopolysaccharides (LPS) that signal through TLR4, resulting in IgA production in a T cell-independent manner, that is, in the absence of CD40L-CD40 interaction (Lebman and Edmiston, 1999; Cerutti, 2008).

C57BL6-CD40L deficient mice (C57BL6-*cd40l* ^−/−^) provide a model that has helped to unveil the physiopathology of human HIGM syndrome (Bernal-Reynaga et al., 2013; Lopez-Saucedo et al., 2015). These mice, in the absence of immunisation, have been shown to express elevated or similar serum concentrations of IgM, accompanied by reduced levels of IgG and IgA, and no IgE as observed in humans presenting HIGM syndrome (Renshaw et al., 1994; Bernal-Reynaga et al., 2013). This animal model has been an excellent tool for studying the interactions between the host and human and mouse gastrointestinal pathogens, such as enterotoxigenic *Escherichia coli* and *Citrobacter rodentium* (*C. rodentium*), respectively (Bernal-Reynaga et al., 2013; Lopez-Saucedo et al., 2015). C57BL6-*cd40l* ^−/−^ *C. rodentium* model revealed that after immunization against this bacterial pathogen specific serum IgG concentrations were lower than in WT mice, nevertheless, they were functional as they exhibited a bactericidal effect against *C. rodentium* (Lopez-Saucedo et al., 2015). Compared to WT mice intestinal IgA concentrations in C57BL6-*cd40l* ^−/−^ mice were significantly higher, IgG1 and IgG2 were significantly lower, and IgG3 levels were similar between mouse strains. For these reasons, the present study seeks to compare and evaluate the presence and localization of GCs, IgA-producing cells, and B-cell populations in both gut-associated (PP and MLN) and non-gut-associated SLOs (spleen and ILN) between unimmunised C57BL6-*cd40l* ^−/−^ and WT mice.

## Material and methods

### Mice strains

WT and C57BL6-*cd40l* ^−/−^ mice were derived from colonies of sentinel animals that were screened for common murine intestinal pathogens once a year and intensively for *Escherichia coli*. Pathogen and *E. coli*-free mice of 6-8 weeks of age were used in all the experiments, which were performed accordingly to the institutional animal guidelines for animal care and experimentation (Protocol number: 0070-13, UPEAL-CINVESTAV-IPN).

### Measurements

Spleens, ILN, MLN, and PPs were harvested from WT and C57BL6-*cd40l* ^−/−^ mice. Following removal of fat from the harvested tissues they were rinsed with PBS (pH 7.4), measured (in centimetres) using a Vernier, and photographed using a Canon Rebel 5Ti camera (Huntington, Nueva York, U.S.).

### *In Situ* immunofluorescence

#### Identification of naïve B cells and IgA-positive plasma cells

Spleens, ILN, MLN, and PPs were harvested as before from WT and C57BL6-*cd40l* ^−/−^ mice (6/group), embedded in Tissue Tek (Leica, IL, USA), immersed in liquid nitrogen, and stored at -70°C until use. Frozen organs were sectioned in 5-6 μm with a Leica cryostat (Leica Microsystems) and mounted on Poly-L Lysine-treated glass slides, fixed in cold acetone for 15 min, after air-drying, they were stored at -20°C. Slides were rehydrated with BSA 0.2%, blocked with Power Block reagent (BioGenex, CA, USA) for 15 min, and then incubated for 1 h with primary anti-mouse antibodies: anti-IgD (BD Pharmingen, 553438), anti-IgM (Southern Biotech, 10-20-08), and anti-CD138 (BD Pharmingen, 553712), after washing, all slides were stained with a secondary anti-rat Alexa Fluor-594 (Life Technologies, A21209) for 1 h at room temperature. Then all slides were further stained with anti-IgA biotinylated (eBioscience, 13-5994-82) and incubated with Alexa Fluor-488-labelled streptavidin (Invitrogen, 511223) for 30 min at room temperature. Finally, for DNA staining slides were incubated with DAPI (4’,6-diamidino-2-phenylindole) for 5 min. Slides were washed and mounted in glycerin. All samples were visualized using an Olympus-BX51 microscope/Olympus RFL-T-epifluorescence lamp, coupled to a U-CMAD3-Olympus camera (Olympus Corporation Japan). Images were analysed with Image-Pro Plus 7.0 software (Media Cybernetics, Rockville, USA) and Fiji Image J software (National Institutes of Health, USA).

B-cell populations were identified as follows; naïve B-cells (IgM^+^IgD^+^), activated B-cells (IgM^+^) plasma cells (CD138^+^), IgA-positive cells (IgA^+^), and IgA-positive plasma cells (CD138^+^IgA^+^).

#### Germinal centre identification

GCs were identified within spleens, ILN, MLN, and PPs follicles by IgD-negative B-cells. Slides were processed as above and stained using anti-IgD as a primary antibody, anti-rat Alexa Fluor-594 as a secondary antibody, and stained with DAPI.

### Flow cytometry

#### Spleens, ILN, PPs, MLN cell suspensions

After harvesting spleens, ILN, MLN, and PPs from WT and C57BL6-*cd40l* ^−/−^ mice (6 mice per/group), cell suspensions were obtained by mechanical disruption, blocked for 15 min with Power Block, and stained for 30 min with anti-CD19-BV605 (BD Pharmingen, 563148), anti-CD11b-PerCp (BD Pharmingen, 550993), anti-IgD-PE (eBioscience, 12-5993-83), anti-IgM-FITC (Southern Biotech, 1020-02), and anti-IgA biotinylated. Plasma cells were identified in separated tubes by anti-CD138 (BD Pharmingen, 561070), washed three times at 1500 rpm for 5 min, permeabilised (BD Cytofix/Cytoperm San Jose, CA) for 20 min, and intracellularly stained for 1 h with anti-IgM-FITC, anti-IgG-APC (Southern Biotech, 17-4010-82) and anti-IgA biotinylated. After washing with PBS (pH 7.4), only labelled cell suspensions stained with anti-IgA were incubated with Alexa Fluor-488-labelled streptavidin, for 15 min. Finally, all cell suspensions were washed with PBS and fixed with 1% paraformaldehyde (Sigma-Aldrich, St. Louis, MO), analysed by flow cytometry (BD LSR Fortessa X-20, Franklin Lakes, New Jersey, U.S), and the obtained data analysed by FlowJo software vX.0.8 (Ashland, OR).

B-cell populations were identified as follows: Total B-cells (CD19^+^), naïve B-cells (IgM^+^IgD^+^), IgA-positive B-cells (CD19^+^IgA^+^), B-1 cells (CD19^+^CD11b^+^), total plasma cells (CD19^+^CD138^+^), IgA-positive plasma cells (CD19^+^CD138^+^IgA^+^), IgG-positive plasma cells (CD19^+^CD138^+^IgG^+^) and IgM-positive plasma cells (CD19^+^CD138^+^IgM^+^). The flow cytometry gating strategy is described in **Supplementary Figure 1**.

#### Peritoneal lavage, cell counting and cytospin assays

Mice were injected intraperitoneally with 5 ml of PBS (pH 7.4), the peritoneal area was then massaged to detach cells, followed by removal of the PBS with a syringe (21G x 32mm). Peritoneal lavages were carried out on 6 mice/group and cell stained to identify IgD, IgM, IgA, CD19, CD11b, and CD138 positive cells, was conducted as described above. For the cytospin assay, peritoneal lavage cells were counted using a Neubauer chamber, and calculations were made to have between 30 to 50 thousand cells/100 mL. Then 100mL of the peritoneal lavage was placed into loading chambers and cytospined (Shandon Cytospin 4, Thermo, Walthan, Massachusetts, U.S.) at a speed of 350 rpm for 5 min.

### Percentages and total numbers of cells

To calculate the total number of cells in a cell suspension per mL the Neubauer chamber was used. The total number of each population was established by using the percentages obtained for each population by FlowJo software.

### Statistical analysis

Values are expressed as mean ±, the standard error of the mean (SEM). Statistical significance was calculated by performing an unpaired Student’s t-test comparing WT mice vs C57BL6-*cd40l* ^−/−^ mice. P values < 0.05 were considered statistically significant. GraphPad Prism software ver.8.0.2 (GraphPad Software, San Diego, CA, USA) was used for the statistical analyses.

## Results

### Identification of germinal centres in secondary lymphoid organs of unimmunised WT and C57BL6-*cd40l* ^−/−^ mice

**Figure 1:**
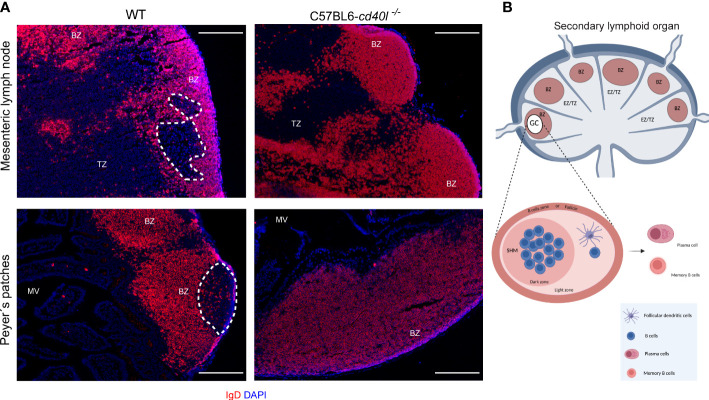
*In situ* distribution of IgD and germinal centre (GC) presence in MLN and PP in unimmunised WT and C57BL6-*cd40l* ^−/−^ mice. **(A)** Naïve B-cells (red) of MLN and PP in WT mice (left panel) and C57BL6-*cd40l* ^−/−^mice (right panel). Cells were stained with DAPI (blue). GCs in MLN and PP are highlighted by the white dotted line, red areas indicate the B-cell zone (BZ), and the extrafollicular zone is stained in blue. GCs were observed in WT mice and were absent in C57BL6-*cd40l* ^−/−^. White bars are equal to 200µm. BZ, B-cells zone; TZ, T-cells zone or EZ, extrafollicular zone and MV, microvilli. Representative images from six independent experiments. **(B)** Scheme of the structure of a secondary lymphoid organ: in red, the B-cell zone is also known as the follicle, in blue, the extrafollicular zone or T-cell zone, and within BZ in white, the GC. The GC magnification denotes the dark zone containing B-cells that undergo somatic hypermutation (SHM), and a light zone harbouring follicular dendritic cells that positively select B-cells. BZ, B-cells zone; TZ, T-cells zone or EZ, extrafollicular zone. Scheme B of **Figure 1** was created using BioRender software (https://www.biorender.com/), agreement number BH25GW7T75.

The presence of GCs within several gut-associated (PP and MLN) and non-gut-associated (spleen and ILN) SLOs of unimmunised WT and C57BL6-*cd40l* ^−/−^ adult mice were examined. After IgD staining (red) to label naïve B-cells and nuclei by DAPI. GCs (white dotted lines) were identified within the PP (**Figure 1A**, left bottom panel) and MLN (**Figure 1A**, left top panel) of WT mice, which are negative for IgD staining, but GC formation was not observed within non-gut associated SLOs in WT mice (**Supplementary Figure 2**). GCs were not observed in all analysed SLOs of C57BL6-*cd40l* ^−/−^ mice (**Figure 1A** right panels and **Supplementary Figure 2** right panels). In both mouse strains, naïve B-cells (IgD-positive cells) were found in the B-cell zone (BZ)

### Presence of IgD- and IgA-positive cells in gut-associated secondary lymphoid organs of unimmunised WT and C57BL6-*cd40l* ^−/−^ mice

**Figure 2:**
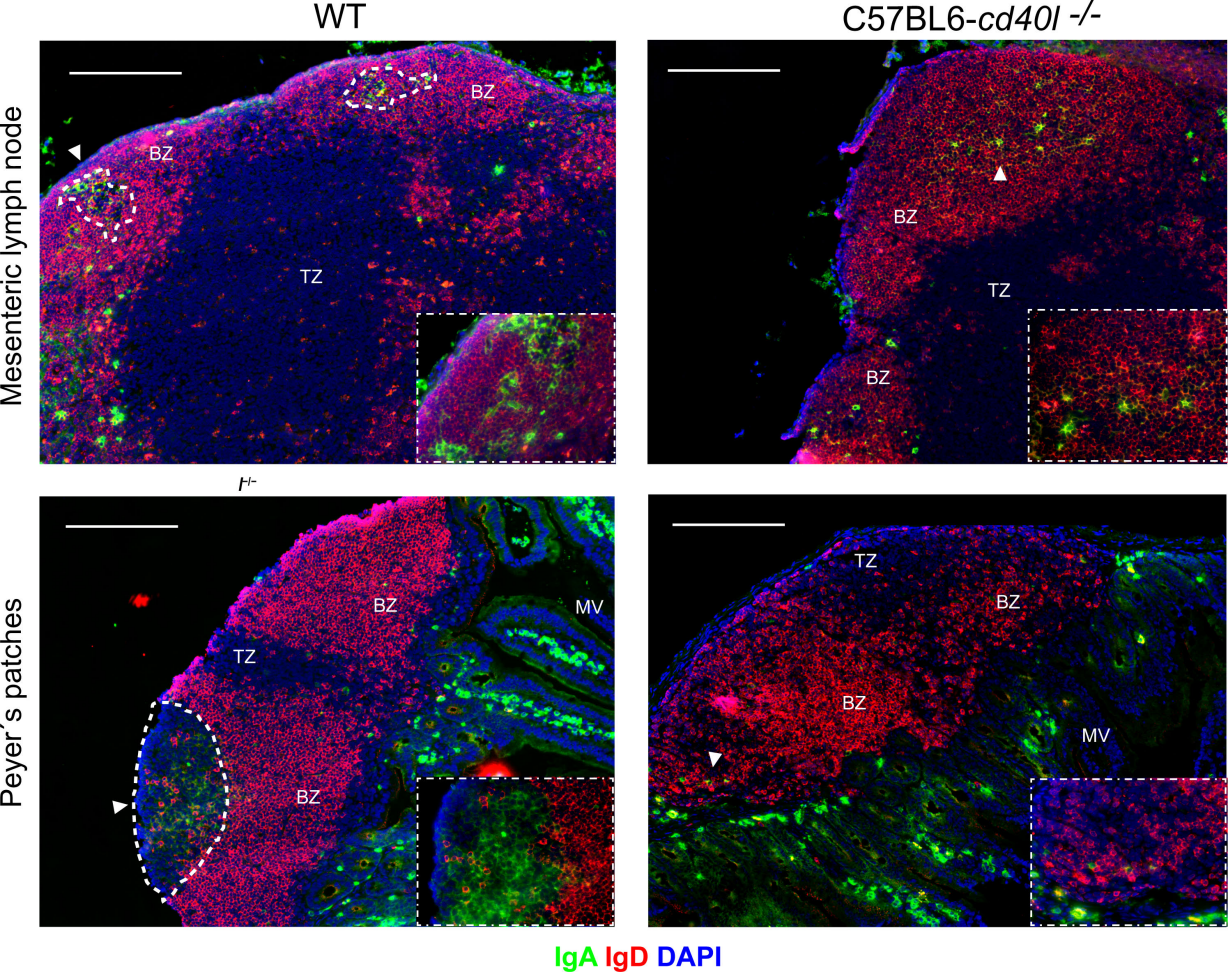
*In situ* distribution of IgD and IgA, among secondary lymph organs in unimmunised WT and C57BL6-*cd40l* ^−/−^ mice. MLN and PP of WT (left panels) and C57BL6-*cd40l* ^−/−^ (right panels) mice, were stained for IgD- (red), IgA-positive cells (green), and DAPI (blue), GCs were highlighted with a dotted white line. In WT mice in both MLN and PP (left panel), all IgA-positive cells were within the GC dotted white line as also shown in the lower right corner square magnification (40X), and all naïve B-cells (red) were outside the GC within the BZ. As shown in the right panel in C57BL6-*cd40l* ^−/−^mice that lack GCs (right panel), IgA-positive cells (white arrows) and B naïve cells (red), were found within the BZ as shown in the lower right corner square magnification (40X). White bars are equal to 200µm. BZ, B-cell zone; TZ, T-cell zone. Representative images from six independent experiments.

After staining MLN and PP for naïve B-cells (red), IgA-positive cells (green), and DAPI (blue) a differential label pattern of IgD- and IgA-positive cells was revealed between mouse strains. In WT mice, in both PP and MLN, naïve B-cells were observed outside the GC area (white dotted lines), while IgA-positive cells were identified within the GCs of these organs (**Figure 2**, left panel). As illustrated in **Figure 2** upper right panel, in C57BL6-*cd40l* ^−/−^ mice, which lack GCs, IgA- and IgD-positive cells were scattered together within the BZ (white arrow), but not in PP where only naïve B-cells were observed within the BZ, plus a few IgA-positive cells were recognized at the TZ in MLN and PP (white arrows) of C57BL6-*cd40l* ^−/−^ mice. Of interest, several IgA-positive cells were observed in the microvilli (MV) in both mouse strains. As shown in **Supplementary Figure 3**, no significant differences were observed between the spleen and ILN for IgD- and IgA-positive cells of WT and C57BL6-*cd40l* ^−/−^ mice.

### Secondary lymphoid organ size, microscopic localisation, and quantification of B-cells including plasma cells in unimmunised WT mice and C57BL6-*cd40l* ^−/−^ mice

#### Spleens

**Figure 3:**
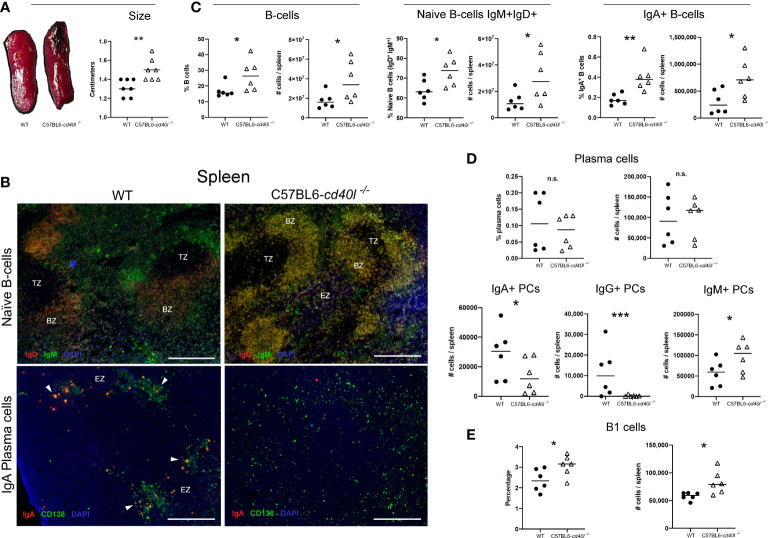
Characterisation and localisation of spleens and splenic B-cell populations, including plasma cells in unimmunised WT and C57BL6-*cd40l* ^−/−^ mice. **(A)** Representative images of WT and C57BL6-*cd40l* ^−/−^ mouse spleens and their length. Overall C57BL6-*cd40l* ^−/−^mouse spleens were significantly bigger P=0.0057, CI (0.06516 to 0.3063) than WT spleens. N=7 per/group. **(B)** Microscopy images of naive B-cells and IgA-positive plasma cells on cryopreserved spleens sections of both mouse strains. Naive B-cells (IgD+ red IgM+ green, merge yellow) in both mouse strains were within the BZ (upper panel). In WT mice splenic plasma cells (green) and IgA-positive plasma cells (yellow/orange) consituted clusters each localized outside the BZ, whereas C57BL6-cd40l−/−mice only plasma cells (green) scaLered around the spleen were observed. White bars are equal to 200μm. BZ, B-cells zone. TZ, T-cells zone. EZ, extrafollicular zone. Representative images from 4 independent experiments. **(C)** Quantification of total B-cells (CD19+), naive B-cells (IgD+IgM+), and IgA-positive B-cellsin the spleens of both mouse strains by flow cytometry. Total B-cells % P=0.045, CI (0.2748 to 19.89) #P= 0.0468, CI (350911 to 40720024), naive B-cells %P= 0.0279, CI (1.252 to 17.58) #P=0.0455, CI (457822 to 36890358), and IgA-positive B-cells %P=0.0054, CI (0.08325 to 0.3667) #P= 0.0308 CI (49407 to 823819) percentages and numbers were significantly higher among C57BL6-cd40l−/−mice in comparison with WT mice. **(D)** Quantification of the total, IgA-, IgG- and IgM-positive plasma cells in the spleens of WT and C57BL6-*cd40l* ^−/−^mice by flow cytometry. The percentages and numbers of total plasma cells were similar between both mouse strains (upper panel). In C57BL6-*cd40l* ^−/−^mice total numbers of IgA- P=0.0452, CI (35163 to 460) and IgG-positive plasma cells P=0.000402, CI (22201 to 621) were significantly lower, and IgM-positive plasma cells P=0.0457, CI (4932 to 84227) were significantly higher, than in WT mice (bottom panel). **(E)** Quantification of total B-1 cells in the spleen of WT and C57BL6-*cd40l* ^−/−^mice. Comparison between C57BL6-*cd40l* ^−/−^mice and WT mice revealed that B-1 cell percentages P=0.0416, CI (0.03276 to 1.381) and total numbers P=0.0194, CI (4958 to 44954) were significantly higher in C57BL6-*cd40l* ^−/−^mice. Flow cytometry data are representative of two independent experiments from 3 mice each. Statistical significance was calculated using an unpaired student´s t-test. Black dots represent WT mice and white triangles represent C57BL6-*cd40l* ^−/−^mice. *P < 0.05, **P < 0.005, ***P < 0.0005. Confidence interval (CL)=95%. n.s. = non-significant.

After measuring (cm) the spleen length it was observed that C57BL6-*cd40l* ^−/−^ mice spleens were significantly bigger (P=0.0057) than WT mice spleens (**Figure 3A**). As illustrated in **Figure 3C**, the comparison between splenic B-cell populations derived from WT and C57BL6-*cd40l* ^−/−^ mice revealed that in C57BL6-*cd40l* ^−/−^ mice the percentage (%) and the number (#) were significantly higher of total B-cells (%P=0.045, #P=0.0468), naïve B-cells (%P=0.0279, #P=0.0455), and IgA-positive B-cells (%P=0.0054, #P=0.0308).

A similar *in vivo* and *in situ* localization of splenic naïve B-cells (IgM^+^ green and IgD^+^ red) was observed in both mouse strains (**Figure 3B**). In WT mice, plasma cells (CD138^+^ green) and IgA-positive plasma cells (CD138^+^ green, IgA^+^ red) were clearly seen forming clusters outside the BZ (white arrows), whereas in C57BL6-*cd40l* ^−/−^ mouse spleens, IgA-positive plasma cells were absent, and only total plasma cells (CD138^+^ green) scattered around the SLO were observed (**Figure 3B**).

As illustrated in **Figure 3D**, percentages and total numbers of spleen plasma cells were similar between both mouse strains (upper panel), but in C57BL6-*cd40l* ^−/−^mice total numbers of IgA- (P=0.0452) and IgG-positive plasma cells (P=0.000402) were significantly lower, and IgM-positive plasma cells (P=0.0457) were significantly higher compared to numbers observed in WT mice (bottom panel).

Since B-1 cells produce IgM plus IgA, and IgG3 antibodies, as well the percentage (P=0.0416), and the total number (P=0.0194) of B-1 cells were compared between WT and C57BL6-*cd40l* ^−/−^ mice demonstrating significantly higher values in the mutant spleens compared to numbers observed for the WT controls (**Figure 3E**).

### Inguinal lymph nodes

**Figure 4:**
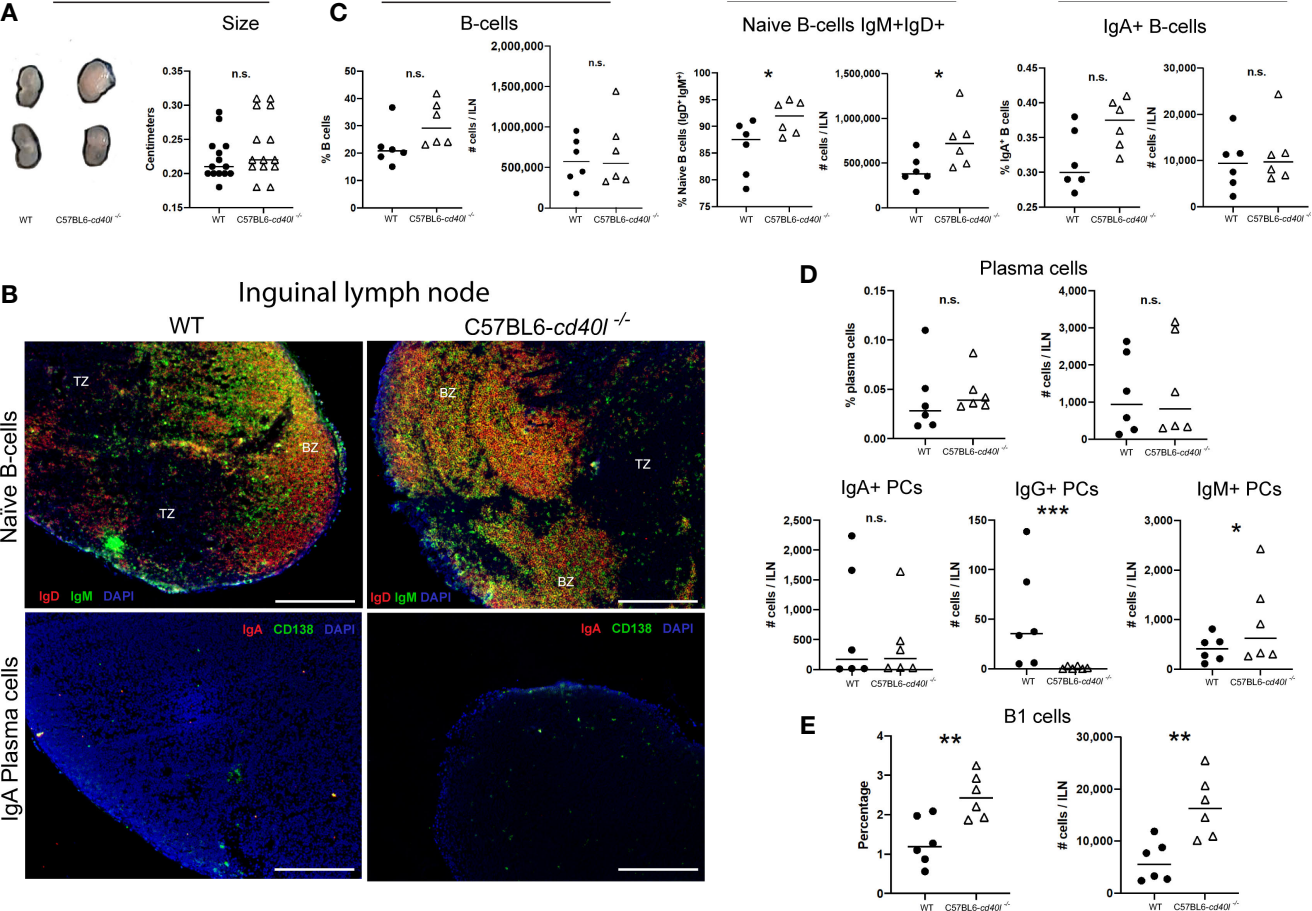
Characterisation and localisation of Inguinal Lymph Nodes (ILN) and B-cell populations, including plasma cells in unimmunised WT and C57BL6-*cd40l* ^−/−^ mice. **(A)** Representative images and length comparison of WT and C57BL6-*cd40l* ^−/−^ mouse ILN. The length of ILN in both mouse strains was similar. N=14 per group. **(B)** Microscopic identification and localization of naïve B-cells and IgA-positive plasma cells on cryopreserved ILN sections of both mice strains. Naïve B-cells (IgD+ red IgM+ green, yellow) in both mouse strains were within the BZ (upper panel). In C57BL6-cd40l−/− mice and WT mice, there were very few plasma cells (green) and IgA-positive plasma cells were absent (yellow/orange). White bars are equal to 200μm. BZ, B-cells zone; TZ, T-cells zone. Representative images from 4 independent experiments. **(C)** ILN quantification and comparison between B-cell populations of both mouse strains by flow cytometry. Naïve Bcells (IgD+IgM+) percentages P=0.0428, CI (0.2265 to 11.24), and numbers P=0.0431, CI (12443 to 651845) were significantly higher among C57BL6- cd40l−/− mice in comparison with WT mice (middle panel). There were no significant differences in the percentage and the total numbers of B-cells (CD19+) and IgA-positive B-cells between C57BL6-cd40l−/− mice and WT mice. (LeH and right panel). **(D)** Quantification and comparison in ILN of plasma cells (total, IgA-, IgG- and IgM-positive) of WT mice and C57BL6-*cd40l* ^−/−^ mice by flow cytometry. The percentages and numbers of total plasma cells were similar between both mouse strains (upper panel). ILN total number of IgA-positive plasma cells was similar between mouse strains (left bottom panel), but in C57BL6-*cd40l* ^−/−^ mice in comparison with WT mice, IgG-positive plasma cells P=0.00025, CI (101.9 to 28.98) and IgM-positive plasma cells P=0.0442, CI (129.8 to 1396) were significantly lower and higher, respectively (middle and right, bottom panel). **(E)** Total number and percentages of B-1 cells in ILN of WT and C57BL6-*cd40l* ^−/−^ mice. Percentages P=0.0063, CI (0.4100 to 1.913) and total numbers P=0.0046, CI (4058 to 16941) of B-1 cells were significantly higher in C57BL6-*cd40l* ^−/−^ mice than in WT mice. Flow cytometry data are representative of two independent experiments from 3 mice each. Statistical significance was calculated using an unpaired student´s t-test. Black dots represent WT mice and white triangles represent C57BL6-*cd40l* ^−/−^ mice. *P < 0.05, **P < 0.005, ***P < 0.0005. Confidence interval (CL)=95%. n.s. = non-significant.

As illustrated in **Figure 4**, panel A, ILN length (cm) was similar in both mouse strains. *In situ* and *in vivo* identification and localisation of naïve B-cells (IgM^+^ green and IgD^+^ red) and IgA-positive plasma cells (CD138^+^ green, IgA red) in ILN tissue sections are described in **Figure 4B**. In both mouse strains, naïve B-cells (yellow) were observed within the BZ (upper panel), in ILN tissue IgA-positive plasma cells (yellow) were not observed at all (bottom panel), plus very few plasma cells (green) were identified at the BZ and TZ (bottom panel).

ILN analysis of total B-cells and IgA-positive B-cells by flow cytometry revealed that the percentages and numbers were similar between both mouse strains (**Figure 4C** left and right), whereas percentages (P=0.0428) and numbers (P=0.0431) of naïve B-cells were significantly higher in C57BL6-*cd40l* ^−/−^mice in comparison with WT mice (**Figure 4C**).

**Figure 4D** (upper and left bottom panels) indicates that in ILN the percentages and total numbers of total plasma cells and IgA-positive plasma cells were similar among both mouse strains. In ILN of C57BL6-*cd40l* ^−/−^mice, the total number of IgG-positive plasma cells was significantly lower (P=0.00025) (middle bottom panel) and the total number of IgM-positive plasma cells was significantly higher (P=0.0442) in comparison with ILN WT mice (right bottom panel).

As shown in **Figure 4E**, in ILN of C57BL6-*cd40l* ^−/−^mice B-1 cells percentages (P=0.0063), and numbers (P=0.0046) were significantly higher than in WT mice.

### Mesenteric lymph nodes

**Figure 5:**
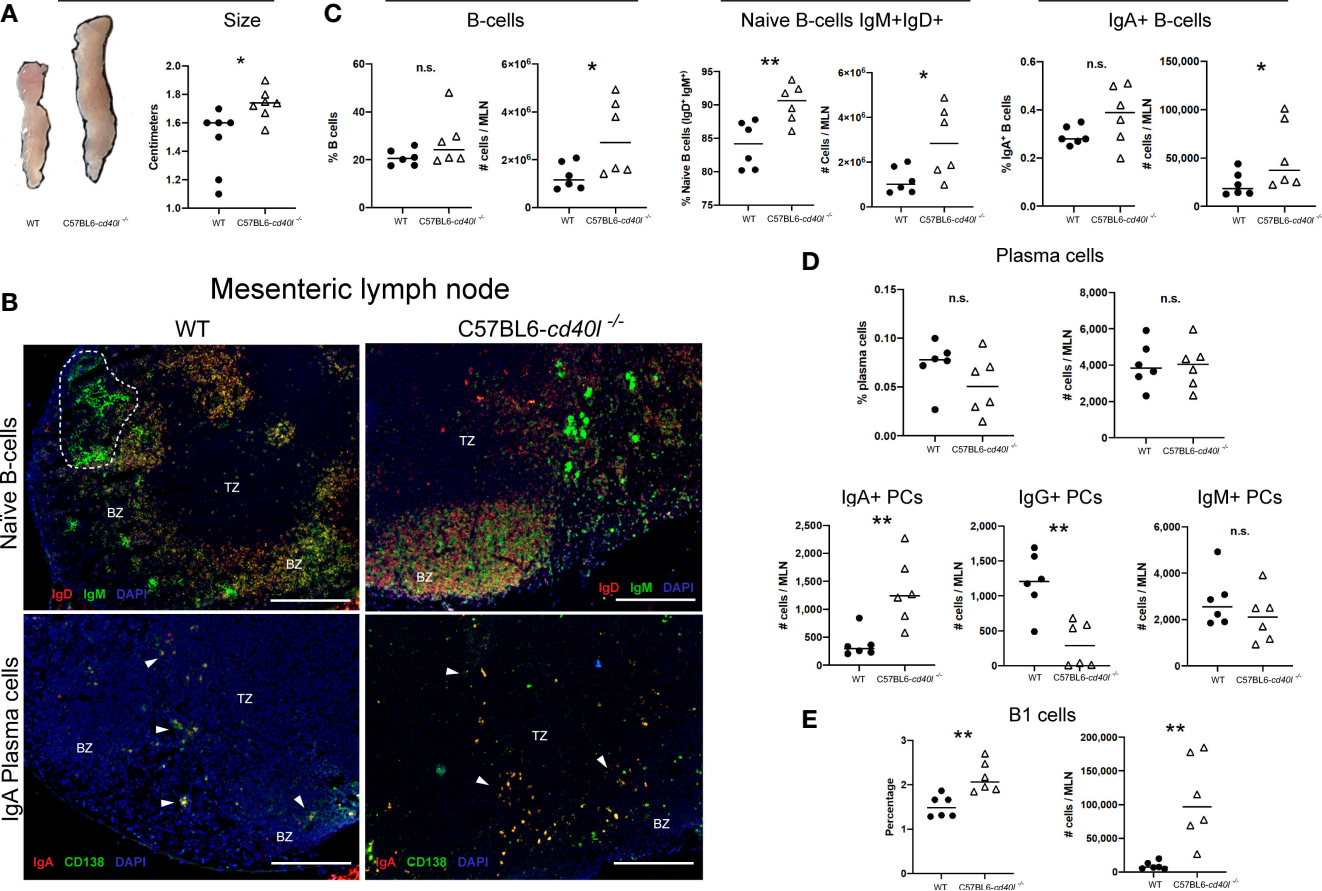
Characterisation and localisation of mesenteric Lymph Nodes (MLN) and B-cell populations, including plasma cells in unimmunised WT and C57BL6-*cd40l* ^−/−^ mice. **(A)** Representative images and length comparison of WT and C57BL6-*cd40l* ^−/−^ mouse MLNs. Overall C57BL6-*cd40l* ^−/−^ mouse MLNs were significantly larger. P=0.0182, CI (0.05292 to 0.4699) than WT MLNs. N=7 per group. **(B)** Microscopic identification and localization of naïve B-cells and IgA-positive plasma cells on cryopreserved MLN sections of both mice strains. Germinal centres (GCs, white doLed lines) were identified in WT mice, but GCs were not identified in MLN sections of C57BL6- cd40l−/− mice. In both mouse strains, naïve B-cells (IgD+ red IgM+ green, yellow) were observed within the BZ (upper panel), and plasma cells (CD138+ green) and IgA-positive plasma cells (CD138+ green, IgA+ red), were mainly located in the TZ (white arrows). In MLN of C57BL6-cd40l−/− mice, it seems that the number of IgA-positive plasma cells (yellow) was higher than in MLN WT mice. White bars are equal to 200μm. BZ, B-cells zone; TZ, T-cells zone. Representative images from 4 independent experiments. **(C)** B-cells (CD19+), naïve B-cells (IgD+IgM+), and IgA-positive B-cells quantification and comparisons between MLNs of both mouse strains by flow cytometry. Naïve B-cell percentages P=0.0071, CI (2.136 to 10.46) and numbers P=0.0332, CI (166632 to 3263944). were higher in C57BL6-cd40l−/− mice and as were the numbers of total B-cells P=0.0377, CI (113232 to 3158067), and IgA-positive B-cell numbers P=0.044, CI (4699 to 62975) compared to numbers observed from WT mice. **(D)** Quantification and comparison of plasma cells (total, IgA-, IgG- and IgM) in MLN of WT and C57BL6-*cd40l* ^−/−^ mice by flow cytometry. The percentages and numbers of total plasma cells were similar in both mouse strains (upper panel). In C57BL6-*cd40l* ^−/−^ mice, total numbers of IgA-positive plasma cells were significantly higher P=0.0048, CI (363 to 1543) and IgG-positive plasma cells significantly lower P=0.0024, CI (1372 to 396), in comparison with WT mice. IgM-positive plasma cells were similar in both mouse strains (bottom panel). **(E)** Total number and percentages of B-1 cells in MLN of WT and C57BL6-*cd40l* ^−/−^ mice. Percentages P=0.0035, CI (0.2729 to 1.047) and total numbers P=0.0033, CI (41356 to 156631) of B1 cells were significantly higher in C57BL6-*cd40l* ^−/−^ mice than in WT mice. Flow cytometry data are representative of two independent experiments from 3 mice each. Statistical significance was calculated using an unpaired student´s t-test. Black dots represent WT mice and white triangles represent C57BL6-*cd40l* ^−/−^ mice. *P < 0.05, **P < 0.005. Confidence interval (CL)=95% n.s. = non-significant.

After comparing the MLN length (cm) of C57BL6-*cd40l* ^−/−^ and WT mice, it was observed that the MLN length of C57BL6-*cd40l* ^−/−^ was significantly longer (P=0.0182) than WT mice (**Figure 5A**).

Naïve B-cell (IgM^+^ green and IgD^+^ red) localization and distribution, *in vivo* and *in situ*, was evaluated using cryopreserved MLN sections of both mouse strains. GCs were identified in WT mice (white dotted lines) while GCs were absent in the MLN of C57BL6-*cd40l* ^−/−^mice (**Figure 5B**). Plasma cells (CD138^+^ green) and IgA-positive plasma cells (CD138^+^ green, IgA^+^ red) in both mouse strains were mainly located in the TZ (white arrows), even though the number of both plasma cells populations was not quantified it seems that the number of these cells is greater in C57BL6-*cd40l* ^−/−^mice than in WT mice (**Figure 5B**).

Flow cytometry analysis of MLN of both mouse strains revealed that percentages of total B-cells and IgA-positive B-cells populations were similar, whereas the total numbers of these two cell populations were significantly higher (P=0.0377 and P=0.044, respectively) in C57BL6-*cd40l* ^−/−^ mice. In MLNs of C57BL6-*cd40l* ^−/−^ mice the total number (P=0.0332) and percentages (P=0.0071) of naïve B-cells were significantly higher than in MLN of WT mice (**Figure 5C**).

As illustrated in the graphs of **Figure 5D**, in MLN of both mouse strains the percentages and numbers of total plasma cells and the numbers of IgM-positive plasma cells were similar (upper and right bottom panels). In C57BL6-*cd40l* ^−/−^ mice MLN the numbers of IgA-positive plasma cells were significantly higher (P=0.0048), whereas the numbers of IgG-positive plasma cells were significantly lower (P=0.0024) than WT mice MLN (bottom panel).

B-1 cells, percentages (P=0.0035), and total numbers (P=0.0033) in MLN suspensions of C57BL6-*cd40l* ^−/−^ mice were significantly higher than in MLN WT mice by flow cytometry (**Figure 5E**).

### Peyer´s patches

**Figure 6:**
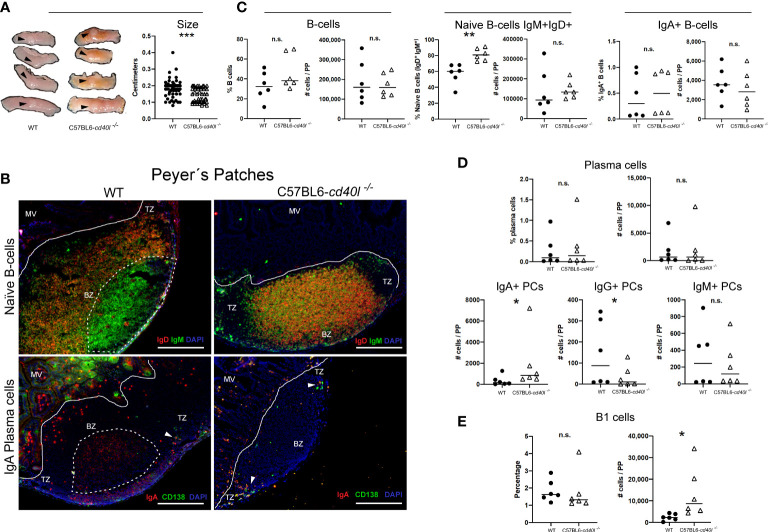
Characterisation and localisation of Peyer patches (PP) and B-cell populations, including plasma cells in unimmunised WT and C57BL6-*cd40l* ^−/−^ mice. Panel **(A)** Representative images and length of WT and C57BL6-*cd40l* ^−/−^ mice PP. Overall C57BL6-*cd40l* ^−/−^ mice PPs were significantly smaller P=0.0002, CI (0.05055 to 0.02470) than the WT PPs. N=56 per/group (7 mice per group). Panel **(B)** Microscopic identification and localization of naïve B-cells (IgD^+^IgM^+^ yellow) and IgA-positive plasma cells (yellow) on cryopreserved PP sections of both mouse strains. In WT mice the presence of germinal centres (GCs, white dotted lines) was observed (upper left panel), which was full of IgM-positive cells. GCs were absent in C57BL6-*cd40l* ^−/−^ mice (upper right panel). In the PP of both mice, a very low number of plasma cells (green) and IgA-positive plasma cells (yellow) (white arrows) were observed and most of them were within the TZ (bottom panel). Within the microvilli, (structure outside the PP region) of WT mice, several plasma cells (green) and IgA-positive cells (red) were observed. In the microvilli of C57BL6-*cd40l* ^−/−^ mice a low frequency of IgA-positive plasma cells (yellow) was observed, and plasma cells (green) were absent. White bars are equal to 200µm. MV, microvilli; BZ, B-cells zone; TZ, T-cells zone. Representative images from 4 independent experiments. Panel **(C)** Quantification and comparison of B-cells (CD19^+^), naïve B-cells (IgD^+^IgM^+^), and IgA-positive B-cells of PP of both mouse strains by flow cytometry. Percentages and numbers of total B-cells and IgA-positive B-cells were similar in both mouse strains. In C57BL6-*cd40l* ^−/−^ mice PP suspensions compared with WT mice PP suspensions percentages of naïve B-cells were significantly higher P=0.0027, CI (10.64 to 37.93). Panel **(D)** Quantification and comparison of plasma cells (total, IgA-, IgG- and IgM-positive) in PP of WT and C57BL6-*cd40l* ^−/−^ mice by flow cytometry. The percentages and numbers of total plasma cells were similar between both mouse strains (upper panel). In C57BL6-*cd40l* ^−/−^ mice total numbers of IgA-positive plasma cells were significantly higher P=0.0496, CI (79 to 3330), IgG-positive plasma cells significantly lower P=0.0415, CI (282 to 19) than in WT mice, and IgM-positive plasma cells were similar between the PP suspensions of both mouse strains (bottom panel). Panel **(E)** Total number and percentages of B-1 cells in PP of WT and C57BL6-*cd40l* ^−/−^ by flow cytometry. The total number of B-1 cells was significantly higher P=0.0411, CI (550 to 21921) in C57BL6-*cd40l* ^−/−^. Flow cytometry data are representative of two independent experiments from 3 mice each. Statistical significance was calculated using an unpaired student´s t-test. Black dots represent WT mice and white triangles represent C57BL6-*cd40l* ^−/−^ mice. *P < 0.05, **P < 0.005, ***P < 0.0005. Confidence interval (CL)=95%. n.s. = non-significant.

PPs of C57BL6-*cd40l* ^−/−^ mice were significantly smaller (P=0.0002) than WT mice PPs (**Figure 6A**).

As illustrated in **Figure 6B**, naïve B-cells (IgM^+^ green and IgD^+^ red) were observed in the follicle of cryopreserved sections of PPs of WT and C57BL6-*cd40l* ^−/−^ mice. GCs were only present (white dotted lines) in WT mice and were full of IgM-positive cells (green) (upper left B panel). In C57BL6-*cd40l* ^−/−^ mice, GCs were absent in PP, naïve B-cells (yellow) were the most prevalent cells within the BZ, and few IgM-positive cells (green) were identified in the TZ (upper right B panel).

As shown in **Figure 6B**, in both mouse strains, a very low number of plasma cells (CD138^+^ green) and IgA-positive plasma cells (CD138^+^ green, IgA^+^ red) were identified in PP TZ (white arrows). In the intestinal cryosection of the WT mouse (left bottom panel), it was possible to identify IgA-positive GCs (red cells, within the dotted lines), IgA-positive cells (red), and several plasma cells (green) were also observed within the MV structures outside the PP region. In C57BL6-*cd40l* ^−/−^ mice, few IgA-positive plasma cells (yellow) and no plasma cells (green) were observed within the MV (right bottom panel).

As illustrated in **Figure 6C**, flow cytometric analysis of PP of both mouse strains indicated that there were no significant differences in the percentages and numbers of total B-cells and IgA-positive B-cells (left and right panels). In C57BL6-*cd40l* ^−/−^ mice there was a significantly higher percentage of naïve B-cells (P=0.0027) than in WT mice (**Figure 6C**).

Similar percentages and total numbers of plasma cells and IgM-positive plasma cell numbers were observed. In PP suspensions of both mouse strains (**Figure 6D**). The number of IgA-plasma cells was significantly higher (P=0.0496), while the number of IgG-positive plasma cells was significantly lower (P=0.0415) in C57BL6-*cd40l* ^−/−^ mice than in WT mice (**Figure 6D**).

Even though the percentages of B-1 cells were similar between mouse strains (**Figure 6E**), the number of B-1 cells in PP suspensions of C57BL6-*cd40l* ^−/−^ was significantly higher (P=0.0411) than in WT mice PP suspensions.

### B-cell and plasma cell populations in peritoneal lavage suspensions of unimmunised C57BL6-*cd40l* ^−/−^ and WT mice

**Figure 7:**
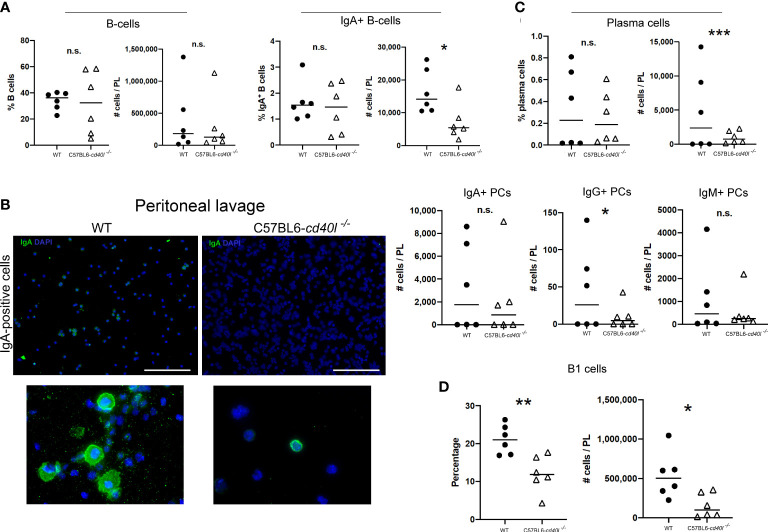
Characterisation of B-cell populations, including plasma cells in peritoneal lavage suspensions of unimmunised WT and C57BL6-*cd40l* ^−/−^ mice. **(A)** Quantification and comparison of total B-cells (CD19^+^), and IgA-positive B-cells in cell suspensions of peritoneal lavages of both mouse strains, by flow cytometry. Percentages and numbers of total B-cells were similar between both mice strains. Total numbers of IgA-positive B-cells were higher P=0.0254, CI (17244 to 1408) in C57BL6-*cd40l* ^−/−^ mice than in WT mice. **(B)** Microscopic identification of IgA-positive cells (green) on peritoneal lavage suspensions by cytospin of both mouse strains. IgA-positive cells in C57BL6-*cd40l* ^−/−^ mice peritoneal lavage suspensions were less abundant than in WT mice (upper panel). The bottom panel illustrates the magnification of the upper panel clearly indicating IgA-positive cells (green). White bars are equal to 200µm. Representative images from 4 independent experiments. **(C)** Quantification and comparison of plasma cells (total, IgA-, IgG- and IgM) in peritoneal lavages suspensions of WT and C57BL6-*cd40l* ^−/−^ mice by flow cytometry. In peritoneal lavage suspension of C57BL6-*cd40l* ^−/−^ mice, the total numbers of plasma cells P=0.000393, CI (9314 to 288) and the numbers of IgG-positive plasma cells P=0.0457, CI (88 to 5) were significantly lower than in WT mice peritoneal lavages. Similar numbers of IgA- and IgM-positive plasma cells were identified in peritoneal lavage suspensions of both mouse strains. **(D)** Total number and percentages of B-1 cells in peritoneal lavage suspensions of C57BL6-*cd40l* ^−/−^ mice and WT. In C57BL6-*cd40l* ^−/−^ mice percentages P=0.0049, CI (14.61 to 3.437) and total numbers P=0.0172, CI (680175 to 83643) of B-1 cells were significantly lower in comparison with WT mice. Flow cytometry data are representative of two independent experiments from 3 mice each. Statistical significance was calculated using an unpaired student´s t-test. Black dots represent WT mice and white triangles represent C57BL6-*cd40l* ^−/−^ mice. *P < 0.05, **P < 0.005, ***P < 0.0005. Confidence interval (CL)=95%. n.s. = non-significant

As illustrated in **Figure 7A**, the percentages, and the numbers of total B-cells (CD19-positive cells) in peritoneal lavages were similar in both animal strains, whereas the number of IgA-positive B-cells (P=0.0254) in C57BL6-*cd40l* ^−/−^ mice peritoneal lavages were significantly lower, in comparison with WT mice.

Microscopic analysis of B-cell populations in the lavage suspensions of both mouse strains, exhibited that in the peritoneal lavages of C57BL6-*cd40l* ^−/−^ mice, IgA-positive cells were less abundant than in peritoneal lavages of WT mice (**Figure 7B**).

The total numbers of plasma cells (P=0.000393) and the numbers of IgG-positive plasma cells (P=0.0457) were significantly lower in peritoneal lavage suspensions of C57BL6-*cd40l* ^−/−^ mice, than in peritoneal lavages of WT mice. Similar numbers of IgA- and IgM-positive plasma cells in peritoneal lavage suspensions of both animal strains were detected (**Figure 7C**).

As shown in **Figure 7D**, peritoneal lavages of C57BL6-*cd40l* ^−/−^ the percentages (P=0.0049) and the total numbers (P=0.0172) of B-1 cells were significantly lower compared to WT mice peritoneal lavages (**Figure 7D**).

### Evaluation of transforming growth factor beta receptor 1 on the surface of total B-cells of unimmunised WT and C57BL6-*cd40l* ^−/−^ mice

**Figure 8:**
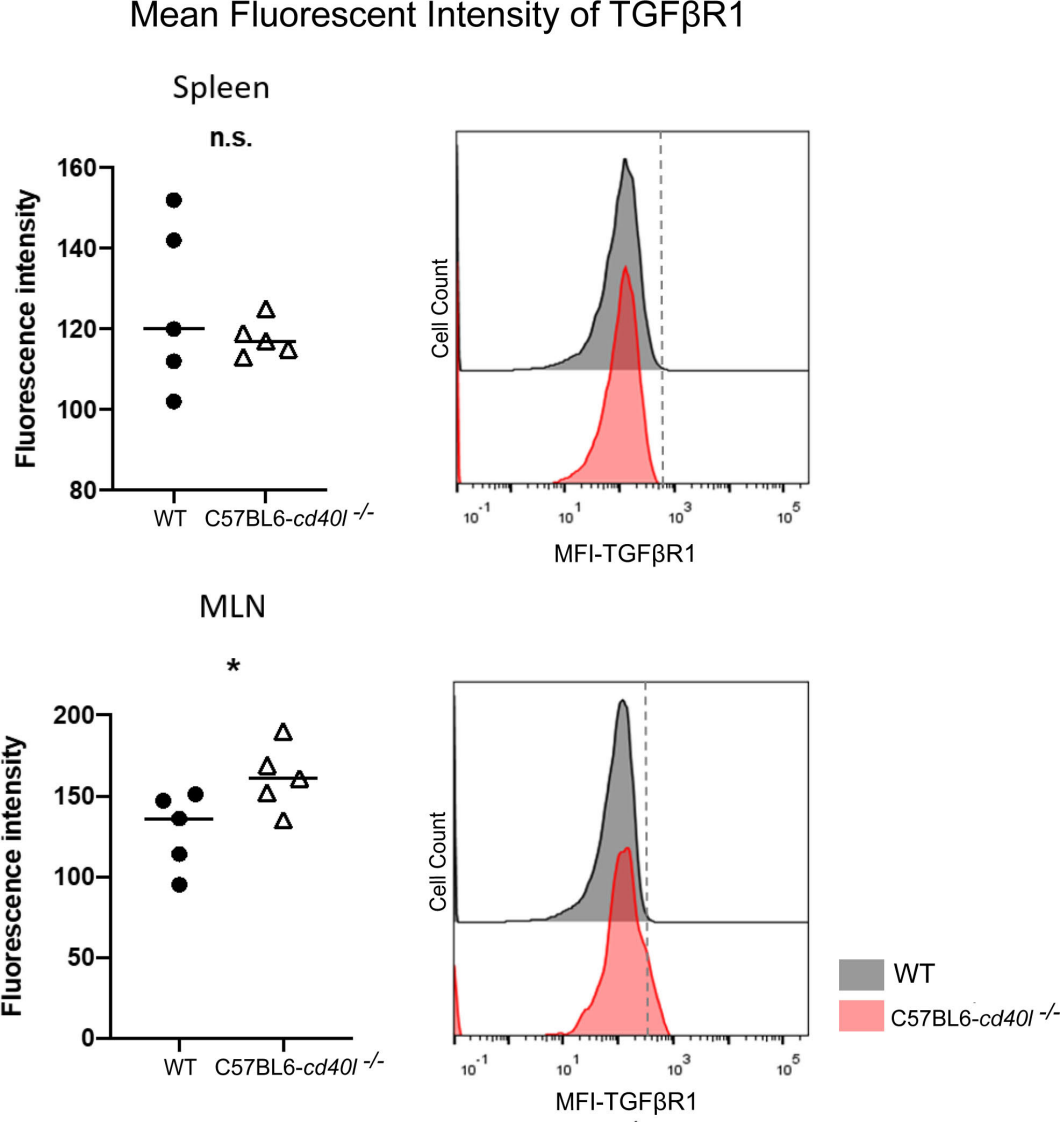
Expression of Transforming growth factor beta receptor 1 (TGFβR1) on the membrane of splenic B-cells and MLN B-cells in unimmunised WT and C57BL6-*cd40l* ^−/−^ mice. **(A)** Comparison of the mean fluorescence intensity of TGFβR1 on splenic B-cells of WT and C57BL6-*cd40l* ^−/−^ mice, by flow cytometry. The receptor membrane expression of TGFβR1 was similar between both mice strains. The right histogram illustrates the fluorescence intensity of TGFβR1 on B-cell membranes of WT (grey) and C57BL6-*cd40l* ^−/−^ mice (red). **(B)** Comparison of the mean fluorescence intensity (MFI) of TGFβR1 on MLN B-cells membrane of WT and C57BL6-*cd40l* ^−/−^ mice by flow cytometry. The expression of TGFβR1 on the membrane of MLN B-cells of C57BL6-*cd40l* ^−/−^ mice was significantly higher P=0.047, CI (0.5585 to 64.96) than the expression on MLN B-cells of WT mice. The right histogram illustrates the fluorescence intensity of TGFβR1 on the B-cells membrane of WT (grey) and C57BL6-*cd40l* ^−/−^ mice (red). Flow cytometry data are representative of 3 independent experiments with a total of 5 mice per/group. Statistical significance was calculated using an unpaired student´s t-test. Black dots represent WT mice and white triangles represent C57BL6-*cd40l* ^−/−^ mice. *P< 0.05. Confidence interval (CL)=95%. n.s. = non-significant.

As illustrated in **Figure 8**, after flow cytometry analysis of the TGFβ receptor 1 (TGFβR1) on splenic B-cells, revealed that the receptor membrane expression was similar among both mouse strains; albeit the expression of TGFβR1 on the membrane of MLN B-cells of C57BL6-*cd40l* ^−/−^ mice was significantly higher (P=0.047) compared to MLN B-cells of WT mice.

### Evaluation of IgA-positive cells in the small intestine of unimmunised WT and C57BL6-*cd40l* ^−/−^ mice

**Figure 9:**
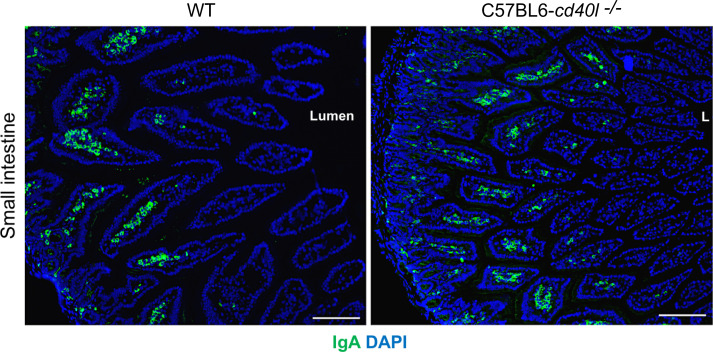
IgA-positive cells in unimmunised WT and C57BL6-*cd40l* ^−/−^ mice small intestine. IgA-positive cells (green) in the small intestine microvilli (MV) of WT (left panel) and C57BL6-*cd40l* ^−/−^mice (right panel), cells were stained with DAPI (blue). MV IgA-positive cell frequency was similar between mouse strains. White bars are equal to 200µm. L, lumen; MV, microvilli. Representative images from 4 independent experiments.

After evaluating in small intestine cryopreserved sections of both animal strains the presence, frequency, and localization (within the microvilli) of IgA-positive cells were similar (**Figure 9**)

## Discussion

The importance of the HIGM syndromes in six Latin-American countries (Argentina, Brazil, Chile, Costa Rica, Peru, and México) was described in 2014, revealing that most HIGM patients (94.5%) have mutations in the *cd40l* gene. The authors also described that the bacteria *Salmonella* spp, the fungus *Microsporidium* spp, and the protozoa *Criptosporidium parvum*, *Giardia lamblia*, *Entamoeba histolytica* and *Isospora belli*, are the most common microorganism associated with chronic diarrheal among HIGM patients in Latin-America (Cabral-Marques et al., 2014).

We described for the first time that in unimmunised C57BL6-*cd40l* ^−/−^ mice exhibit significantly higher concentrations of IgA in their faeces in comparison with WT mice (Bernal-Reynaga et al., 2013). IgA is the most abundant immunoglobulin in the intestinal tract, which derives from B-cells, receiving stimulating signals from intestinal microorganisms, in a T-dependent and T-independent fashion. Mice gut IgA responses to intestinal bacteria contribute to shaping bacterial topography, composition, growth, invasiveness, and even modulating the host immunometabolic functions, and immune responses (Chen et al., 2020). Therefore, to understand the observation of higher concentrations of gut IgA in unimmunised C57BL6-*cd40l* ^−/−^ mice, we determined the prevalence of several B-cell populations and plasma cells (total, IgA-, IgG- and IgM-positive plasma cells) in gut-associated (MLN and PP) and non-gut-associated SLOs (spleen and ILN) in WT mice and C57BL6-*cd40l* ^−/−^ mice.

In unimmunised WT mice, GCs were present in-gut associated SLOs but absent in non-gut-associated SLOs, this could be due to the fact of the continuous antigen stimulation of intestinal SLOs by microorganisms and food antigens, as it is well established (Forchielli and Walker, 2005; Van den Broeck et al., 2006; Suzuki et al., 2010a; Stebegg et al., 2018). In line, it has also been described that after WT mice were orally immunised with ovalbumin-cholera toxin, IgA-positive cells were absent or present in low numbers within the spleen and other non-gut associated lymph nodes, respectively (Weiberg et al., 2018), also indicating that antigen SLOs stimulation is influenced by immunisation site (Drayson, 1986). In contrast, in PP and MLN of WT mice a higher frequency of IgA-positive cells within the GC, known as IgA-GC, was observed, as has been shown in antigen-driven reactions (Mora and von Andrian, 2008). IgA-positive cells were observed within gut-associated SLOs of C57BL6-*cd40l* ^−/−^ mice even in the absence of GC, revealing a differential abundance and localization of IgA-positive cells, which were more abundant and located within the BZ in MLN, and less abundant and located within the TZ in PP. Overall our results suggest that in unimmunised mice GC formation and production of IgA-positive cells, are mainly limited to gut-associated SLOs that are constantly exposed to microbial and food antigens (Suzuki et al., 2010b).

As previously described in C57BL6-*cd40l* ^−/−^ mice GCs were absent in non-gut-associated (Spleen and ILN) SLOs, and we described for the first time that GCs are also absent in gut-associated SLOs (PP and MLN) of C57BL6-*cd40l* ^−/−^ mice. Furthermore, CD40 receptor-deficient C57BL6 mice also lacked GCs in gut-associated (PPs and MLN) SLOs (Castigli et al., 1994; Bergqvist et al., 2006). Together these results confirm that the interaction between CD40 (on B-cells) and CD40L (on activated T-cells) is essential for the formation of GCs (Kawabe et al., 1994; Renshaw et al., 1994; Takemori et al., 2014).

The characterisation of IgA-positive cells *in vivo* and *in situ* in non-gut- and gut-associated SLOs in both unimmunised mouse strains, revealed that in non-gut-associated SLOs (spleen and ILN) IgA-positive cells were identified within the TZ, and their frequency was very low. In contrast, a high and similar number of IgA-positive cells were observed in the small intestine MV of WT and C57BL6-*cd40l* ^−/−^ mice. Confirming that most intestinal IgA production does not require CD40-CD40L interaction, GC formation, and arises from a T-independent manner (Fleming et al., 2022).

In C57BL6-*cd40l* ^−/−^ mice the total numbers of IgM-positive plasma cells were significantly higher, while IgA-positive plasma cells were significantly lower at all non-gut-associated SLOs studied, in comparison with WT mouse SLOs; corroborating that C57BL6-*cd40l* ^−/−^ mice are producing a higher or equal number of serum IgM and lower IgA antibodies (Bernal-Reynaga et al., 2013) comparable to HIGM patients (Meng et al., 2018).

Contrary to gut-associated SLOs in C57BL6-*cd40l* ^−/−^ mice IgA-positive plasma cells were significantly higher compared to gut-associated SLOs of WT mice, consistent with the previous report of higher concentrations of IgA in the faeces of C57BL6-*cd40l* ^−/−^ mice than in WT mice (Bernal-Reynaga et al., 2013). It has also been reported that IgG in faeces of C57BL6-*cd40l* ^−/−^ mice are significantly lower than in WT mice (Bernal-Reynaga et al., 2013); in line, we observed that SLOs IgG-positive cells frequency was very low or absent in C57BL6-*cd40l* ^−/−^ mice than in WT mice, confirming that most IgG responses take place in GCs (Elsner and Shlomchik, 2020). Together our results confirm the role of PP and MLN as mucosal inductive sites, whose characteristic features are to initiate an IgA preferential immune response production in these anatomical sites (Koch et al., 2016; Weiberg et al., 2018). IgA antibodies play a pivotal role in neutralizing, eliminating, and regulating potential pathogens and microorganisms in the gut (Gommerman et al., 2014; Takeuchi and Ohno, 2021).

Furthermore, we have shown that immunized C57BL6-*cd40l* ^−/−^ mice with *C. rodentium*, have complement-mediated bactericidal effect on *C. rodentium*, and indeed was observed in the sera of C57BL6-*cd40l* ^−/−^ mice contained IgM and IgG2b antibodies that are capable of fixing complement. (Lopez-Saucedo et al., 2015); hence, it is possible that intestinal IgM and IgG will also have a bactericidal effect against intestinal bacterial pathogens in C57BL6-*cd40l* ^−/−^ mice (Lopez-Saucedo et al., 2015), but the presence of IgG2b in faeces must be shown.

B-1 cells, which are an important source of secreted IgA and IgM natural antibodies, in intestinal and pulmonary mucosae, were as well characterised in peritoneal lavages and SLOs of both animal strains. (Suzuki et al., 2010a; Baumgarth, 2011; Prieto and Felippe, 2017). Notably, B-1 cells that have not been previously characterised in the peritoneal lavage of C57BL6-*cd40l* ^−/−^ mice, were significantly less abundant than in WT mice peritoneal lavages, but were more abundant within the gut and non-gut associated C57BL6-*cd40l* ^−/−^ mouse SLOs than in WT mouse SLOs. Indicated that in C57BL6-*cd40l* ^−/−^ mice, B-1 cells are migrating to immune effector sites, all around the mouse body, in order to produce natural antibodies (Hardy and Hayakawa, 2005). It has been shown that B-1 cells and their antibodies play an important role in the protection against mice pneumococci illness (caused by *Streptoccocus pneumoniae*), animals lacking B-1 cells were unable to survive pneumococci infections, may be due to the fact that natural IgM and IgA are not produced, especially against the pneumococcal capsular polysaccharide serotype 3 (PPS)-3, a non-protein antigen, found on the cell walls of these bacteria (Dasu et al., 2009; Rodriguez-Zhurbenko et al., 2019). Also, the human B-1 cell population generates antibodies against the capsular polysaccharides of *S. pneumoniae* and polysaccharide Vi of *Salmonella typhi* (causative agent of typhoid fever) suggesting the important role of the B-1 population in mucosal immune responses in animals and humans (Suzuki et al., 2010b; Marshall et al., 2012; Rodriguez-Zhurbenko et al., 2019).

Since TGFβ superfamily members contribute to the maturation, and differentiation of B-cells, particularly promoting IgA production by inducing the differentiation of B-cells to IgA-producing plasma cells (Li et al., 2006; Tamayo et al., 2018; Takeuchi and Ohno, 2021). After evaluating the presence of TGFβ receptor 1 (TGFβR1) on splenic B-cells of both mouse strains a similar expression of this receptor was observed. In contrast, TGFβR1 expression on B-cells of MLN, a gut-associated SLOs, was significantly higher in C57BL6-*cd40l* ^−/−^ mice compared to MLN B-cells of WT mice. These observations together with the fact that in C57BL6-*cd40l* ^−/−^ mice the number of IgA-positive cells and B-1 cells in MLN, were significantly higher than in WT mice, suggest that the microenvironment in MLN is promoting the induction of IgA plasma cells that in turn will produce gut IgA antibodies in C57BL6-*cd40l* ^−/−^ mice.

Together our results may explain our initial observation of a higher concentration of IgA antibodies in C57BL6-*cd40l* ^−/−^ mice faeces in the absence of GCs. Additionally, it was described that IgA-positive cells, IgA-positive plasma cells, B-1 cells, and B-cells TGFβR1 are more abundant in the gut-associated SLOs of C57BL6-*cd40l* ^−/−^ mice than in SLOs of WT mice. The B-cell populations identified in gut-associated SLOs and the microenvironment (due to overexpression of TGFβR1) are contributing in C57BL6-*cd40l* ^−/−^ mice to increase the production of total IgA antibodies in the gut. The high concentration of intestinal IgA constitutes a barrier for the adhesion, growth, elimination, and/or clearance of intestinal microbial pathogens in mice and in HIGM patients.

## Conclusion

Most HIGM patients (95%) have mutations of the *cd40l* gene, patients are characterised by higher/similar IgM and lower IgG, and IgA serum concentrations and by the absence of GC in SLOs. C57BL6-*cd40l* ^−/−^ mouse, has serum immunoglobulin concentrations parallel levels observed in HIGM patients and lacks GC in SLOs, thus is an animal model mimicking HIGM patient characteristic. Our group has reported that C57BL6-*cd40l* ^−/−^ mice have lower IgG and higher IgA faecal antibodies than WT mice and specific serum (IgM and Ig2b) antibodies against *C. rodentium* that have a complement-mediated bactericidal effect on these bacteria. Using the C57BL6-*cd40l* ^−/−^ mouse model under unimmunised conditions, it was revealed that all SLOs tested (Slpleen, ILN, MLN, and PP) lacked GCs and contained higher numbers of B-1 cells, all gut-associated SLOs harboured higher numbers of IgA-positive plasma cells that WT mice, whereas both mouse strains have similar expression of IgA-positive cells in small intestine lamina propria. IgA-positive plasma cells and B-1 cells are directly involved in the production of IgA antibodies in the intestine. Our recently acquired knowledge will help to develop specific treatments for the elimination of harmful pathogens in the intestine of HIGM patients, like priming the intestine with pathogens antigens that in turn will induce the production of specific gut IgA, IgM, IgG2b, and IgG3 antibodies.

## Data availability statement

The original contributions presented in the study are included in the article/**Supplementary Material**. Further inquiries can be directed to the corresponding authors.

## Ethics statement

The animal study was reviewed and approved by CINVESTAV Ethics Committee, # 254994.

## Author contributions

LF-R, TE-G, and FH-C conceived the experiments. FH-C, RM-A, and CL-S performed the experiments. FH-C worked on the quality of the images and figures.JY-P, JM-B, and SE-P provided guidance, resources, and input. FH-C and TE-G wrote the manuscript, which was reviewed and approved by all authors. All authors contributed to the article and approved the submitted version.

## Supplementary material

Supplementary Figure 1
*In situ* distribution of IgD and germinal centres (GCs) presence in the spleen and inguinal lymph node (ILN) of WT and C57-cd40l−/− at the steady state. Naïve B cells (red staining) of the spleen and ILN in WT mice (left panel) and C57-cd40l−/− mice (right panel). Cells were stained with DAPI (blue), red areas indicate the B cell zone (BZ), and the extrafollicular zone is stained in blue. GCs were absent in WT mice and C57-cd40l−/−mice. White bars are equal to 200µm. BZ, B cells zone; TZ, T cells zone or EZ, extrafollicular zone. Representative images from six independent experiments.

Supplementary Figure 2
*In situ* distribution of IgD and IgA, in the spleen and inguinal lymph node (ILN) of WT C57-cd40l−/− mice. The spleen and ILN of WT (left panel) and C57-cd40l−/− (right panel) mice, were stained for IgD- (red), IgA -positive cells (green), and DAPI (blue), GCs were absent in the spleen and ILN of both mice strains. A very few IgA-positive cells were present in the spleen and ILN of WT and C57-cd40l−/− mice. White bars are equal to 200µm. BZ, B cell zone; TZ, T cell zone. Representative images from six independent experiments.
