# Supplementary figures and images for: Elevated levels of enteric IgA in an unimmunised mouse model of Hyper IgM syndrome derived from gut-associated secondary lymph organs even in the absence of germinal centres
Source: Front Cell Infect Microbiol. 2023 Jun 29;13:1172021. doi: 10.3389/fcimb.2023.1172021 (PMC10339347; doi:10.3389/fcimb.2023.1172021)

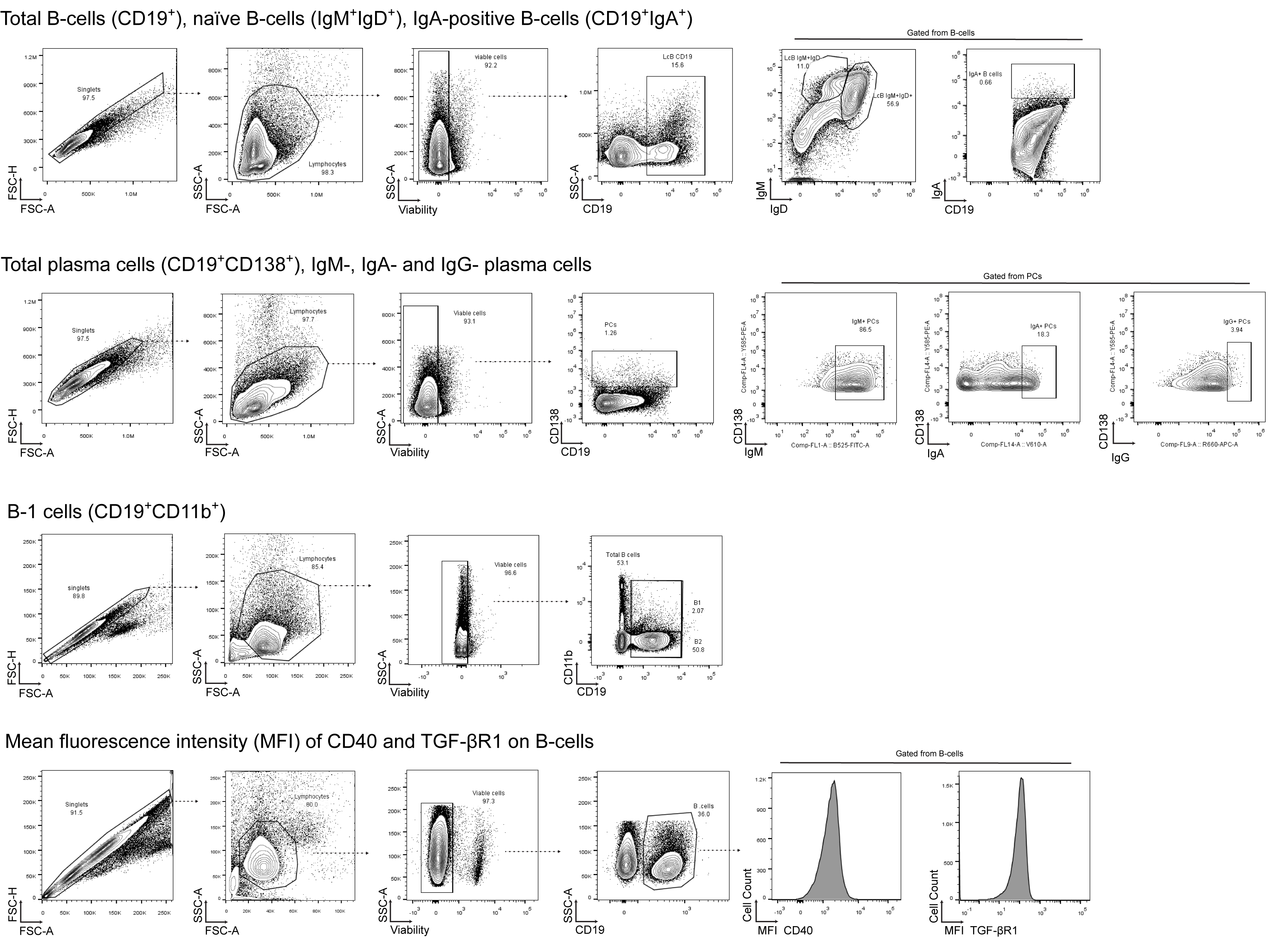

Supplement: Supplementary Figure 1 — In situ distribution of IgD and germinal centres (GCs) presence in the spleen and inguinal lymph node (ILN) of WT and C57-cd40l−/− at the steady state. Naïve B cells (red staining) of the spleen and ILN in WT mice (left panel) and C57-cd40l−/− mice (right panel). Cells were stained with DAPI (blue), red areas indicate the B cell zone (BZ), and the extrafollicular zone is stained in blue. GCs were absent in WT mice and C57-cd40l−/−mice. White bars are equal to 200µm. BZ, B cells zone; TZ, T cells zone or EZ, extrafollicular zone. Representative images from six independent experiments. [file Image_1.tif]

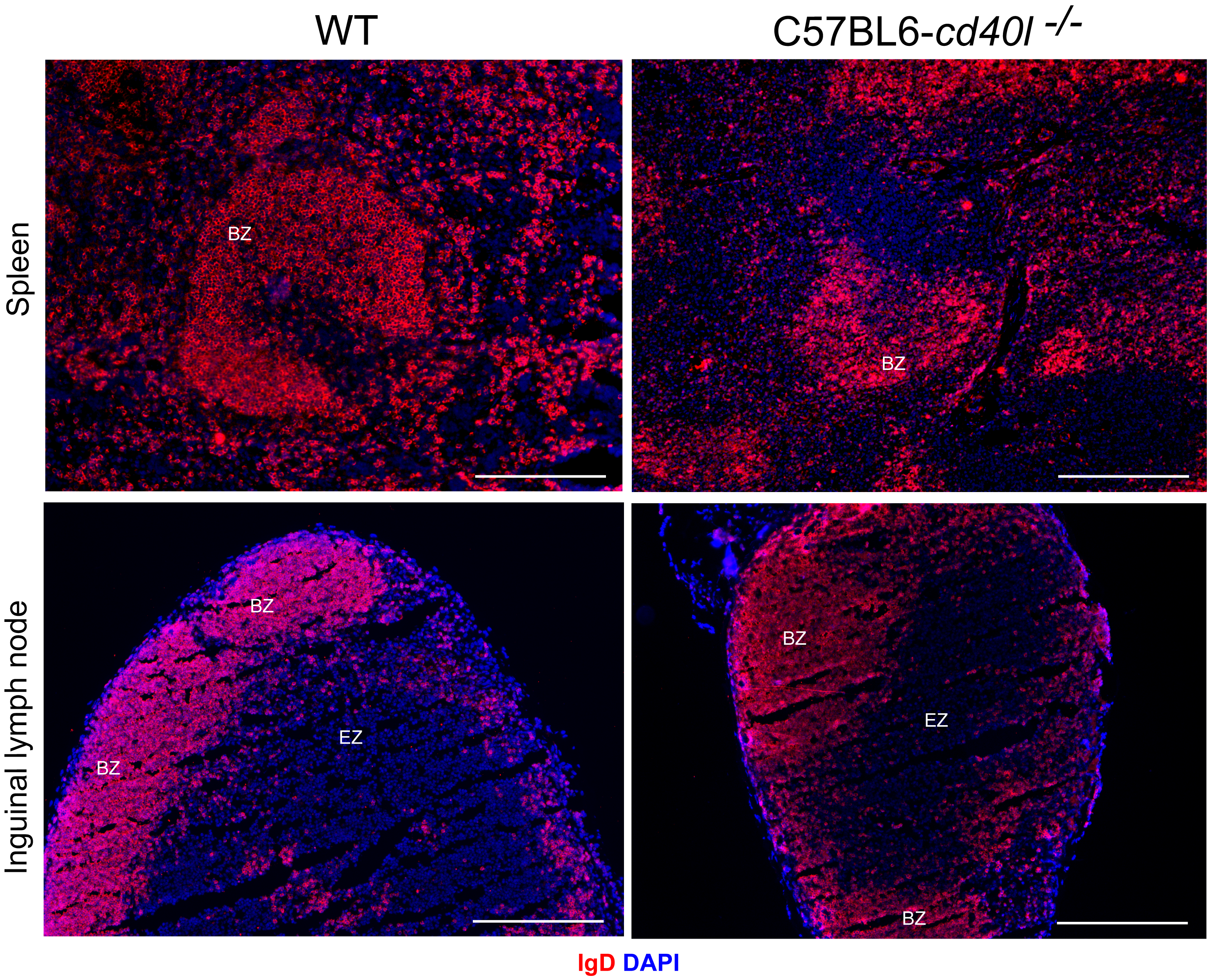

Supplement: Supplementary Figure 2 — In situ distribution of IgD and IgA, in the spleen and inguinal lymph node (ILN) of WT C57-cd40l−/− mice. The spleen and ILN of WT (left panel) and C57-cd40l−/− (right panel) mice, were stained for IgD- (red), IgA -positive cells (green), and DAPI (blue), GCs were absent in the spleen and ILN of both mice strains. A very few IgA-positive cells were present in the spleen and ILN of WT and C57-cd40l−/− mice. White bars are equal to 200µm. BZ, B cell zone; TZ, T cell zone. Representative images from six independent experiments. [file Image_2.tif]

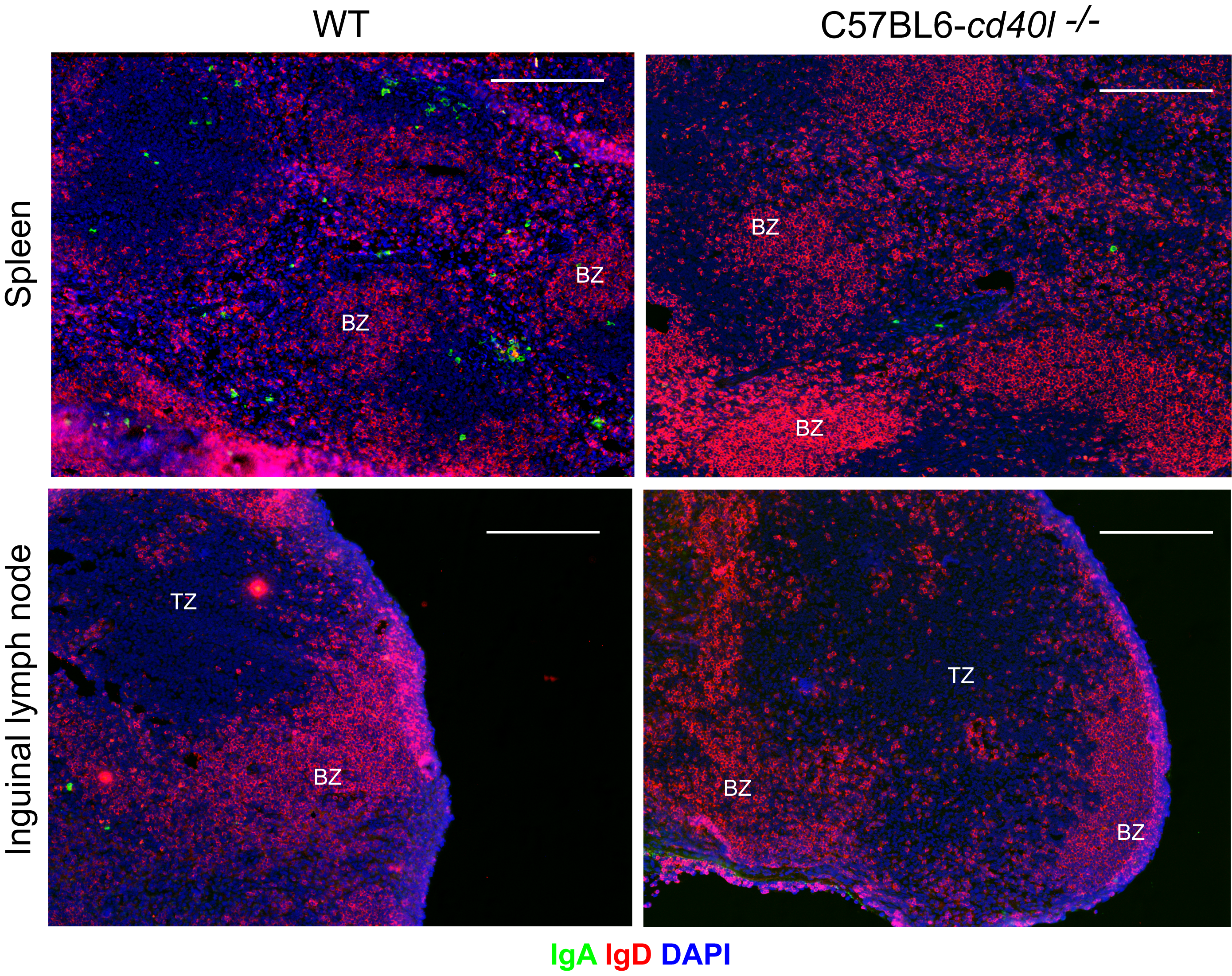

Supplement: Supplementary file 3 [file Image_3.tif]
